# Pediatric heart failure with preserved ejection fraction, a review

**DOI:** 10.3389/fped.2023.1137853

**Published:** 2023-08-02

**Authors:** Sophie Quennelle, Damien Bonnet

**Affiliations:** ^1^ Pediatric Cardiology Department, Necker-Enfants Malades Hospital, Paris, France; ^2^ Equipe Projet HeKA, Paris, France; ^3^ Université Paris Cité, Paris, France

**Keywords:** heart failure with preserved ejection fraction, diastolic heart failure, pediatric, child, cardiomyopathy, pulmonary hypertension

## Abstract

Diastolic dysfunction refers to a structural or functional abnormality of the left ventricle, resulting in impaired filling of the heart. Severe diastolic dysfunction can lead to congestive heart failure even when the left ventricle systolic function is normal. Heart failure with preserved ejection fraction (HFpEF) accounts for nearly half of the hospitalizations for acute heart failure in the adult population but the clinical recognition and understanding of HFpEF in children is poor. The condition is certainly much less frequent than in the adult population but the confirmatory diagnosis of diastolic dysfunction in children is also challenging. The underlying causes of HFpEF in children are diverse and differ from the main cause in adults. This review addresses the underlying causes and prognostic factors of HFpEF in children. We describe the pulmonary hypertension profiles associated with this cardiac condition. We discuss diagnosis difficulties in clinical practice, and we provide a simplified diagnostic algorithm for HFpEF in children.

## 1. Introduction

Left Ventricular (LV) diastolic function plays an important role in determining the kinetics and quality of ventricular filling and thereby also the stroke volume. It can be defined as the capacity of the heart to relax and distend during diastoles leading to fulfilling of the LV chamber up to an appropriate telediastolic volume. Diastolic dysfunction is defined by the incapacity of the ventricle to fill in even if the atrial pressure is normal. Diastolic dysfunction might be due to a prolonged relaxation or/and an increased stiffness of the ventricle (1). The normal functioning of the left ventricle during diastole involves the relaxation of its walls, allowing for the ventricle to expand and accommodate incoming blood. This is facilitated by the normal functioning of muscle cells within the ventricle, which contract during systole and then relax during diastole. However, in diastolic dysfunction, the relaxation of the ventricular walls is impaired or slowed, resulting in decreased filling of the left ventricle. At a cellular level, relaxation of the left ventricle during diastole is enabled by the detachment of actin-myosin cross bridges, which occurs after calcium uptake by the sarcoplasmic reticulum, leading to a decrease in cellular tension. It is the main protein that's modulates myocyte stiffness. Additionally, the increased stiffness of the ventricular walls, due to conditions such as fibrosis or hypertrophy, can also contribute to diastolic dysfunction by limiting the ventricle's ability to expand and accommodate blood. Chamber stiffness is primarily determined by the stiffness properties of the sarcomeres, the interstitial space, and LV chamber geometry and wall thickness (1). Another component that influences LV filling and diastolic pressures is elastic recoil. Early diastolic recoil, is the ability of the left ventricle to “snap back” into its relaxed state, it contributes to the quick decrease in LV pressure during isovolumic relaxation time (IVRT). In diastolic dysfunction, this recoil may be delayed or reduced, further impeding the ventricle's ability to fill with blood during diastole. Titin plays an important role in LV expansion. It is an elastic protein that assembles sarcomere unity and acts as an elastic spring that is squeezed during systole and recoils during diastole (2). Left atrial pressure, is closely related to LV pressure and is used as a surrogate marker of LV filling pressures. During diastole, when the LV is filling with blood, the pressure within the LV pressure increases. This increased pressure is then transmitted backwards into the left atrium, resulting in an increase in left atrial pressure. Therefore, in conditions where LV diastolic function is impaired, there is an associated increase in LAP, reflecting the elevated filling pressures within the LV.

Today, the commonly used definition is heart failure with a left ventricle ejection fraction (LVEF) >50% associated with evidence of spontaneous or provokable increased left ventricle (LV) filling pressures (2). There is a thin line between HFpEF and diastolic dysfunction as patients with LV diastolic dysfunction are often asymptomatic at rest but experience limitations during exercise. Clinical presentations of HFpEF rank from acute heart failure to shortness of breath during effort. While adults are able to describe the onset or worsening of shortness of breath after having experienced well-tolerated efforts in the past, poor exercise tolerance in a child could be difficult to identify.

## 2. Epidemiology

LV diastolic dysfunction has been well studied and described in adults as HFpEF accounts for at least half the patients diagnosed with heart failure (3). Risk factors in adults include age, obesity, hypertension, coronary artery disease and diabetes mellitus (4, 5). However, comprehensive studies were conducted on elderly diabetic and hypertensive patients (6–8) and do not reflect diastolic dysfunction epidemiology and physiopathology in the pediatric population. Indeed, insights on the epidemiology and causes of HFpEF in children are scarce. There are few studies that evaluated the LV diastolic function in children (9–11) and diastolic function screening is challenging in routine pediatric cardiologist's evaluation (12). As a consequence, HFpEF prevalence in children is unknown and probably underestimated. Pan et al. (10) reported 421 children suffering from diastolic heart failure. This large cohort was collected between 2004 and 2014 at the Children's Hospital of Chongqing Medical University. Among a series of 3,907 pediatric hospitalizations in a cardiology department that were retrospectively reviewed, only 18 patients were considered as having HFpEF (0.5%) (11).

## 3. Physiopathology

HFpEF is a heterogeneous group of diseases characterized by symptoms of heart failure due to increase in myocardial stiffness. The physiology and cellular mechanism of HFpEF depends on the cause and involves, among other things, cardiac hypertrophy and fibrosis (13), intrinsic myocardial dysfunction (14) impaired calcium handling (15), titin stiffening (16), inflammatory state (17), large and small vessels disease (18).

In restrictive cardiomyopathy (RCM), LV wall thickness is more often within normal limits and for Gewillig the pathophysiology of RCM is related to myocyte abnormality such as myocyte hypertrophy, enlarged, irregular and hyperchromatic myocyte nuclei (19). Genetic disorders leading to intracellular accumulation of substances such as Anderson Fabry disease (20), Danon disease (21), iron overload cardiomyopathy (22) are examples of myocyte induced restrictive physiology.

In the study of Lombardi et al. (23), a linear correlation between aortic elasticity and LV diastolic function was observed, which raises the hypothesis that, just like in the elderly population, aortic vascular stiffness leads to LV diastolic dysfunction. Indeed, with increased vascular stiffness, the systolic pulse wave velocity is increased and, thus, the reflected wave returns to the heart earlier, during late systole increasing late systolic afterload, which affects thick-thin myofilament interactions and crossbridge dissociation, leading to impaired relaxation.

Interstitial myocardial fibrosis also plays a role in the pathogenesis of the LV diastolic dysfunction as it increases ventricular rigidity and compromise the stretching capability of the myofibrils (24). Collagen is a key component of the myocardial extracellular environment, and increased collagen deposition alters myocardium viscoelasticity, impairing relaxation, diastolic recoil, and passive stiffness (25). Fibrosis is a common endpoint of myocardium pathologic processes such as chronic ischemic heart disease of myocarditis.

## 4. Etiology

The causes of LV diastolic dysfunction in children differs substantially from the classical risk factors identified in adults, intrinsic myocytes abnormality seems to be the leading cause of diastolic dysfunction in children. Primary cardiomyopathies are diseases of the heart muscle in which the myocardial dysfunction appears in the absence of systemic comorbidities, valvular or congenital heart disease (26). Hypertrophic, dilated, and restrictive cardiomyopathies are primary cardiomyopathies that can lead to diastolic dysfunction. There are two major clinical categories of LV diastolic dysfunction in children: congenital, related to a genetic or metabolic disorder and acquired i.e., secondary to another cause (27). In this review, we choose not to describe transitory diastolic dysfunction related to congenital heart defect as it almost always resolves after cardiac surgery.

### 4.1. Congenital: genetic hypertrophic, restrictive cardiomyopathies, inherited infiltrative cardiomyopathies

Dilated cardiomyopathy (DCM) is defined by the presence of a dilated LV with systolic dysfunction without any hemodynamic, physiological, ischemic or anatomic cause (28). DCM accounts for around 50% of the pediatric cardiomyopathy cases (26). Patients suffering from DCM present at first heart failure with reduced ejection fraction, but progression of cell death and extension of fibrosis of the LV often lead to diastolic dysfunction. Occurrence of diastolic dysfunction with elevated filling pressures is associated with poor prognosis in these conditions (29).

Hypertrophic cardiomyopathy (HCM) is defined by an hypertrophied and non-dilated ventricle in the absence of a hemodynamic cause of wall thickening, excluding physiological hypertrophy (secondary to physical activity) and pathological hypertrophy (secondary to aortic coarctation, aortic valvular stenosis, hypertension) (28). HCM is the second most common pediatric cardiomyopathy (40% of patients) (26). Diastolic function impairment is common and of variable severity. Impaired calcium handling, myocyte hypertrophy and disarray, ischemia and fibrosis are the etiologies of diastolic dysfunction (30).

Diastolic dysfunction has been described mainly in children suffering from restrictive cardiomyopathy, accounting for less than 5% of pediatric cardiomyopathy cases (19, 26). Leading mutations associated with restrictive cardiomyopathy are MYH7, TNNI3, TNNT2, MYL2, and DES (31). Restrictive cardiomyopathies may also be secondary to systemic diseases including infiltrative disorder, collagen-vascular diseases (9), neoplastic process (32), thoracic irradiation and chemotherapy, bone marrow transplantation (26, 32, 33), and endomyocardial disease with or without eosinophilia resulting in fibrosis of the endocardium. Left heart obstruction such as coarctation and aortic stenosis can result in restrictive physiology of the left ventricle that persists after repair (18, 34, 35). The common mechanism in these cardiomyopathies is the increasing myocardial stiffness, which decreases compliance.

### 4.2. Acquired: relaxation abnormalities secondary to cardiac remodeling and fibrosis reducing relaxation capacity

LV diastolic dysfunction has been described in patients with aortic coarctation or aortic stenosis (18, 23, 24, 35) even after successful repair. In the study of Moskowitz et al. (35), altered LV relaxation was found in children with successful coarctation repair and late diastolic filling pressures were influenced by LV mass suggesting that hypertrophy rather than increased afterload is the primary determinant of the impaired LV diastolic function.

Some extrinsic factors have also been described in children such as inflammatory diseases: acute myocarditis (36), Kawasaki shock syndrome (37), and more recently Multisystem Inflammatory Syndrome in Children (MISC) due to severe acute respiratory syndrome coronavirus 2 (SARS-CoV-2) (38). Graft rejection is also a cause of LV diastolic dysfunction in pediatric heart recipients (39, 40). Myocardial edema secondary to the inflammation causes the impaired ventricular compliance.

Transient LV diastolic dysfunction can be observed in acute contexts such as tachycardia, during the early post-operative period or during sepsis. It is important to note that at birth (during the first 7 days/the first two months for preterms) the diastolic properties of the LV are significantly impaired without pathological significance.

As for adults, renal insufficiency (41–44) and obesity are risk factors for diastolic dysfunction (45, 46). Restrictive physiology of the left ventricle has also been described in infants of mothers with gestational diabetes (47–49). Other acquired conditions include deposition diseases that reduce ventricular compliance (e.g., iron infiltration) (22).

## 5. Clinical presentation

In some cases, diastolic dysfunction may not cause any symptoms and may only be suspected during a routine echocardiography. In other cases, it can cause symptoms such as shortness of breath, atypical asthma, and syncope. The clinical signs that may suggest the presence of diastolic dysfunction include increased jugular venous pressure, hepatomegaly, oedema and ascites (19). Lack of appetite, fatigue and infection susceptibility are markers of heart failure exacerbation. Electrocardiogram (EKG) findings are generally not specific for diastolic dysfunction. Electric signs of left atrial enlargement, biatrial hypertrophy, abnormal Q wave or pseudoinfarct pattern, repolarization abnormalities such as the T wave notched or biphasic can be noted (19). Chest x-ray can show pulmonary venous congestion, and, depending on the underlying cardiac condition, cardiomegaly (19). These symptoms are not specific and may be related to left ventricular systolic dysfunction which makes the diagnosis even more difficult.

## 6. Pulmonary hypertension

Group 2 pulmonary hypertension includes PH associated with left heart disease including HFpEF, heart failure with reduce ejection fraction, valvular heart diseases and congenital heart diseases. Despite similar hemodynamic profiles, there are significant disparities between these patients' groups in terms of comorbidities and clinical phenotypes (50). The hemodynamic definition of group 2 PH was proposed during the 6th World Symposium on PH as a mean pulmonary artery pressure of >20 mm Hg and a pulmonary artery wedge pressure of >15 mm Hg (51). HFpEF associated with PH can be subdivided into isolated postcapillary pulmonary hypertension (IPH) and combined postcapillary and precapillary pulmonary hypertension (CPH). The diagnosis is based on the pulmonary vascular resistance (PVR) measurement during RHC: <3 Wood units (WU) for IPH or ≥3 WU for CPH. This distinction is crucial since it influences therapeutic strategies (52). The pathophysiologic mechanisms in HFpEF associated with PH have been well described in adults. Abnormal relaxation and increased stiffness of the LV lead to an increase in LV and left atrial pressure (53). Left atrial elevated pressures expose the lung pulmonary veins to elevation in pressure (54) and cause the stress failure of the alveolar-capillary junction resulting in pulmonary edema and inflammation. Inflammatory mediators increase endothelin-1 depression and decrease nitric oxide and natriuretic peptide activity. The fibroblast proliferation is activated and leads to the occlusion of the lumen and thickening of the alveolar septa (55). This structural remodeling associated with lung edema compresses small lung arterioles and induces precapillary PH. Thus, with progress of the diastolic disease PH can evolve from a postcapillary to a combined PH (pre and postcapillary). The pulmonary hemodynamic profiles in HFpEF have received little attention but the development of PH with elevated pulmonary vascular resistance in HFpEF is recognized as an important contributor to morbidity and mortality. PH profiles has not yet been studied in pediatrics HFpEF patients but PH has been well described in children suffering from RCM as a relevant proportion of them developed CPH unresponsing to vasodilator testing and precluding them from orthotopic heart transplantation (56). Up to half of the RCM children have PH at diagnosis (57) it is a factor of poor prognosis and therapeutic solution are scares (58).

## 7. Diagnostic

The clinical recognition and understanding of heart failure with preserved ejection fraction in children is poor. This might be because it is a rare condition but also because the diagnosis of diastolic dysfunction in children is difficult in clinical practice. The symptoms may be subtle and can vary depending on the severity of the condition. The consensual definition of HFpEF is the presence of signs and symptoms of heart failure, a LV ejection fraction >50% associated with evidence of spontaneous or provokable increased LV filling pressure (3).

The gold standard to characterize LV relaxation is cardiac catheterization. A high-fidelity catheter placed into the LV measure simultaneously LV volumes and pressure. The LV pressure-volume loops reflects the filling and contractile properties of the ventricle (5). The commonly measured parameters are (1) Time constant of relaxation (tau or *τ*) that reflects the rate of decline of LV pressure during the isovolumic relaxation period (IVRT). Normal duration of tau is 30–40 ms (1). (2) Pulmonary capillary wedge pressure (PCWP) is the pressure measured in the pulmonary artery during diastole, which reflects the pressure in the left atrium and left ventricle. (3) Left ventricular end-diastolic pressure (LVEDP) is the pressure measured directly within the left ventricle at the end of diastole. Also, rapid saline loading during RHC can be used to assess the response of the LV and further elucidate the pathophysiology of heart failure (59). Indeed, an increase in PCWP in response to an increased afterload may suggest that diastolic dysfunction is related to increased ventricular stiffness.

Because of its invasive nature, technical complexity, and cost, heart catheterization is impractical for the routine evaluation of the LV filling pressures that are almost always estimated by transthoracic echocardiography (TTE). The H2FPEF (60) and the HFA–PEFF diagnostic algorithm (61) have been proposed to evaluate LV filling pressure in adults but precise thresholds are missing in the pediatric population (12). Indeed, in children, tachycardia and pediatric particularities make Doppler measurements challenging and adults cut-off values for these various parameters are inappropriate (62). Former studies attempted to determine specific TTE parameters to facilitate LV End Diastolic Pressure (LVEDP) estimation (63, 64). Dragulescu and al. found that the mitral E wave deceleration time (DT) (153 ± 23 ms in control patient vs. 97 ± 27 ms in RCM patients) and the mitral lateral peak early diastolic tissue velocity (normal if >11 cm/s) were the most discriminating parameters (12). Peak late diastolic mitral velocities (A) vary significantly throughout childhood, this impacts the peak early to late diastolic filling velocity (E/A) ratio and this parameter should not be considered without taking into account the patient's age (65). In contrast, the isovolumic relaxation time appeared stable with age which makes it an excellent parameter in children. Sasaki and al. compared the echocardiographic findings of 9 RCM patients with 27 controls and concluded that the left atrial size distinguished patients with and without diastolic dysfunction with the least overlap as the indexed left atrial area was significantly larger in RCM group: median 22.8 cm^2^/m^2^ (range 16.9–28.6) vs. 10.3 cm^2^/m^2^ (range 8.3–12.3) in the control group (66). By providing quantitative analysis of LV longitudinal function speckle tracking-derived global longitudinal strain (GLS) is accurate in early detection of subclinical alterations in LV longitudinal function (63). There is not clear threshold for normal GLS in children (64). In the study of McAree and al (67), it appears that patients suffering from MISC presented a lower mean GLS than controls (−20.4 (±2.8) vs. −22.0 (±1.9).

The B-type natriuretic peptide (BNP) and its pro-hormone NT-proBNP (N-terminal pro-B-type natriuretic peptide) are produced by the ventricles in response to increased pressure or volume loads. It is typically released when the myocytes are stretched or under stress (68) NT-proBNP levels can be elevated in children with left ventricular diastolic dysfunction because the increased pressure or volume in the heart leads to the release of NT-proBNP. Higher levels of NT-proBNP can therefore be a marker of diastolic dysfunction and may be used to help diagnose this condition. However, NT-proBNP levels are dependent on age and, to a lesser extent, on gender in the pediatric population. In the review of Nir et al. (69), the data from four studies which included patients who had NT-proBNP dosage for other reasons than heart failure was concatenated, taking the 95th percentile as the upper limit for normal. They proposed the normal NT-proBNP threshold for age (reported in Table 1). Inspired by H2FPEF (60) and the HFA–PEFF diagnostic algorithm (61) that is used in adults, we propose a stepwise diagnosis approach that is a compilation of published criteria (Table 1). Another pertinent biomarker is aldosterone, indeed, in the study of Masutani and al (11). the aldosterone/BNP ratio was markedly higher in HFpEF patients than in HFrEF patients (38 ± 63 vs. 1.7 ± 1.9, *P* < 0.05) and “ROC curve analysis showed that an aldosterone/ BNP ratio of 10.3 or higher best predicted HFpEF [area under the curve (AUC) = 0.89], with a sensitivity of 0.67 and specificity of 1.0.”.

**Table 1:** Stepwise diagnosis approach for HFpEF in children.

Signs and symptoms of heart failure	Dyspnea, fatigue, low exercise tolerance, lack of appetite, infection susceptibility
Suggestive TTE parameters	LVEF > 50%
	No other cause of heart failure (absence of shunt or valvular disease)
	DT < 100 ms
	TRIV < 90 ms
	E’ < 11 cm/s
	LE enlargement > 15 cm^2^/m^2^
	Low GLS
Elevated NT pro-BNP levels (by age)	0–2 days > 12,000 pg/ml
	3–11 days > 6,000 pg/ml
	1 month–1 year > 650 pg/ml
	1–2 years > 400 pg/ml
	2–6 years > 300 pg/ml
	6–18 years > 160 pg/ml (69)
Therapeutic testing	Symptoms improved by diuretics
Right heart catheterization	LVEDP > 15 mmHg.
	If doubtful, a rapid saline loading test may sensitive the RHC

LVEF, left ventricular ejection fraction; DT, mitral E wave deceleration time; IVRT, isovolumic relaxation time; E’ sep, peak early diastolic tissue velocity at medial mitral annulus; LAVi, left atrial volume indexed to body surface area; LVEDP, Left ventricular end diastolic pressure; RHC, right heart catheterization.

Cardiac magnetic resonance imaging (CMRI) is a non-invasive imaging modality that can provide valuable information about the structure and function of the heart. By providing details about myocardial phenotype such as the localization of the myocardial hypertrophy (septal or apical) in HCM or the specific myocardial composition such as the presence of fibrosis, edema, fat, iron overload (by T2* imaging) in patients with hemochromatosis (70) CMRI is helpful to access the etiology of HFpEF. CMRI allows myocardial tissue characterization and fibrosis extension which makes it the modality of choice in diagnosing and following up cardiomyopathies. Indeed, the administration of intravenous gadolinium based contrast is the gold standard to locate and quantify the extension of the myocardial fibrosis (71). In the absence of focal LGE, T1 relaxation times and extracellular volume evaluation confirm the diagnosis of diffuse fibrosis (72). The extend of myocardial fibrosis estimated by late gadolinium enhancement (LGE) significantly correlates with the degree of diastolic dysfunction (25). Quantification of fibrosis, maximum wall thickness, and LV mass are markers of risk for sudden cardiac death that can be obtained by CMRI. Another important feature of CMRI is its ability to provide information on blood flow patterns in the heart. The analysis of LV filling velocity and volume flow, of the volumetric assessment of LV and of left atrium makes CMRI an highly accurate and reproducible noninvasive technique for the assessment of diastolic function (73). The wave intensity represents the energy flux per unit area that is carried by waves traveling in the cardiovascular network and derived from CMRI flow data (74). There are two main wave patterns during systole: the forward compression wave at systolic ejection (FCW) and the forward expansion wave (FEW) at end systole. The FCW/FEW ratio was significantly lower in patients suffering from HFpEF as peak FCW was lower in patients with LV diastolic dysfunction. CMRI is helpful in assessing graft function in pediatric heart transplant recipient (75).

## 8. Clinical course and outcomes

The clinical course of children with diastolic dysfunction varies depending on the underlying cardiac condition. Global prognosis of HFpEF in children is uncertain especially since this term refers to a heterogeneous group of patients. There is limited literature on the prognosis of HFpEF in children beyond the phenotypes of HCM and RCM. No well-defined predictors exist for determining survival after diagnosis but it appears that RCM prognosis is grim due to the risk of sudden death and progression of pulmonary hypertension leading to early transplantation (76), younger age at diagnosis, repeated admissions for heart failure, diuretic use and isolated RCM phenotype are risk factors for death (76, 77). In the National Heart, Lung, and Blood Institute (NHLBI) Pediatric Cardiomyopathy Registry, children with RCM who had congestive heart failure and a lower fractional shortening Z score at diagnosis had the worst prognosis. The occurrence of LV diastolic dysfunction in patients with dilated cardiomyopathy is a marker of poor prognosis (29, 78) but there are no specific studies in children. Masutani et al. (11) compared the clinical course of 18 children suffering from HFpEF to 22 children suffering from HF with reduced EF (HFrEF) and observed that the heart failure mortality rate was significantly lower in the HFpEF than in the HFrEF patients (22% vs. 41%). Future works should focus on patients with isolated left ventricular diastolic dysfunction (and preserved ejection fraction) to define the pertinent predictor of outcome.

## 9. Treatment

It is important for children with left ventricular diastolic dysfunction to receive timely and appropriate treatment to help prevent any long-term complications. To date, there is no pharmaceutical drug facilitating myocardial relaxation or improving ventricular compliance. Also, pharmaceutical treatment for HFpEF in children is extrapolated from adult clinical trials. Lifestyle management in children includes fighting against obesity and poor diet, blood pressure control and physical activity. Medical management strategies include diuretics to relieve symptoms of congestive heart failure. In adults' patients, the precise monitoring of the pulmonary pressures through a device, the CardioMEMS Heart Sensor, allows for efficient diuretic titration and has proven its benefit in reducing hospitalization for heart failure in patients suffering from HFpEF (79, 80). Beta Blockers may be useful as they increase diastolic duration by slowing down the heart rate (81). Spironolactone appears to improve prognosis in obese and diabetic adult patients suffering from HFpEF (15, 82) but these results are not transferable to pediatric patients. Angiotensin-converting enzyme inhibitors and sartans are prescribed with the intention to treat and prevent both systolic and diastolic dysfunction in the case of cardiomyopathies and after aortic coarctation repair but there is no evidence of efficacy of this treatment on the diastolic function. Empagliflozin and sacubitril/valsartan (83) have shown promising results in adults but data on children are not available yet. To date, no therapeutic trial has shown any benefit in treating patients suffering from HFpEF associated with PH with pulmonary arterial hypertension-approved therapies (84). In patients suffering from post-capillary PH and heart failure symptoms, the atrial flow regulator, an interatrial shunt device that recently became available proved to be efficient on symptoms and secure including in the pediatric population (85–87).

## 10. Conclusion

HFpEF is a rare and probably under-diagnosed condition in children. It can be caused by a variety of underlying conditions. Clinical presentation is not specific, and the diagnosis may be difficult. We proposed a diagnosis algorithm easy to use in clinical practice. Prognosis depends on the cause, but the appearance of associated hypertension marks a turning point in the evolution of the disease. Specific treatments are limited.
